# Employing students’ evaluations and tutors’ perceptions to evaluate a faculty development program on problem-based learning at the Faculty of Medicine, King Abdulaziz University

**DOI:** 10.1186/s12909-024-05662-1

**Published:** 2024-07-01

**Authors:** Ahlam Barnawi, Ahmed M. Sonbol, Lana Al-Shawwa, Alwalla Abulaban, Khalil Asiri, Abdulaziz Bagasi, Reem Alafari, Aliaa Amr Alamoudi

**Affiliations:** 1 Department of Respiratory Therapy, Intensive Care Unit, King Fahad General Hospital, Jeddah, Saudi Arabia; 2 Musculoskeletal Centre of Excellence, International Medical Centre, Jeddah, Saudi Arabia; 3 Department of Medical Education, Faculty of Medicine, King Abdulaziz University, Jeddah, Saudi Arabia; 4 Department of Respiratory Therapy, King Abdulaziz Medical City, Jeddah, Saudi Arabia; 5 Bariq Health Sector, Ministry of Health, Asir, Saudi Arabia; 6 Department of Family Medicine, Ministry of National Guard - Health Affairs , Jeddah, Saudi Arabia; 7 Clinical Biochemistry Department, Faculty of medicine, King AbdulAziz University, Jeddah, Saudi Arabia

**Keywords:** Problem-based learning, Evaluation, Faculty development program, Kirkpatrick model

## Abstract

**Background:**

Faculty development programs are crucial for promoting continuous learning, enhancing teaching effectiveness, and encouraging professional growth among medical educators. Problem-based learning was introduced as a teaching strategy in our Faculty of Medicine in 2007. Thereafter, several rounds of a faculty development program were conducted to help teachers recognize their role as facilitators and assess areas for improvement.

**Methods:**

We conducted a mixed-methods study with a sample of 284 third-year medical students answering a questionnaire and 21 faculty members participating in focus groups. A validated 13-item questionnaire was used to investigate the students’ evaluation of their tutors’ performance in problem-based learning. Three sessions were then conducted with faculty members involved in problem-based learning to gain in-depth insights into their experiences and perspectives.

**Results:**

The mean performance ranking for tutors awarded by the students was above halfway. There was a significant positive correlation between tutors’ performance ranking and all five of the learning approaches examined herein: constructive/active learning, self-directed learning, contextual learning, collaborative learning, and intra-personal behavior (*p* < 0.05). The data from the focus groups were analyzed under five broad themes: tutors’ insights into their strengths and weaknesses, challenges in conducting problem-based learning, tutors’ ways of preparing for problem-based learning, feedback, and suggestions for improving problem-based learning workshops.

**Conclusions:**

This study recommends improvements and future directions for advanced program evaluation. Faculty development programs can be tailored to effectively address students and faculty members’ goals and needs, which can benefit the teaching and learning process and foster a culture of continuous improvement and professional growth.

**Supplementary Information:**

The online version contains supplementary material available at 10.1186/s12909-024-05662-1.

## Background

Faculty development programs (FDPs) play a central role in medical education by promoting continuous learning, enhancing teaching effectiveness, and encouraging professional growth among medical educators [25]. McLean et al. [19] defined FDP as “the personal and professional development of teachers, clinicians, researchers, and administrators to meet the goals, vision, and mission of the institution in terms of its social and moral responsibility to the communities it serves.” FDPs offer faculty members valuable opportunities for enhancing their knowledge and refining their instructional skills. By participating in FDPs, educators can improve their ability to deliver education, employ innovative teaching methods, and engage students effectively [28]. These programs also offer them a platform for collaborating and sharing ideas, thereby creating an academic community attentive to excellence in medical education. Overall, FDPs contribute to the development of medical education by making educators well equipped, motivated, and capable of preparing the next generation of healthcare professionals [13]. Some of them also involve initiatives for retaining employees, developing personal and professional responsibility, and orienting new staff [32].

There have been ongoing global commitments to FDPs to improve medical education and meet evolving healthcare needs. In 1966, the World Health Organization [20] published a report entitled “The training and preparation of teachers for medical schools with special regard to the needs of developing countries,” which provided recommendations for an international program of medical teacher training and a framework for subsequent developments in the field. More recently, there have been efforts to improve medical education in the scientifically developed world, with emphasis on adapting to changes in medicine. Several conferences have been held to address the future of faculty development, emphasizing the need for a global perspective and international collaboration. For example, the conference on “A 2020 Vision of Faculty Education Development across the Medical Education Continuum” in 2010 at the Baylor College of Medicine in Houston, Texas aimed to develop recommendations for training faculty who prepare physicians to meet evolving healthcare needs [26]. Additionally, medical schools are increasingly investing in faculty development. There is significant evidence of ongoing commitments to FDPs in the Arabian Gulf as well. Abdulrahman et al. [1] recommended making FDPs a requirement for all new faculty members before they are assigned any academic responsibility. They also recommended that these programs’ levels, activities, content, and timings be devised according to individualized needs assessment. An important recommendation was establishing a medical education department/center/office/unit and special committee for FDPs.

This recommendation materialized at the Faculty of Medicine (FOM) at King Abdulaziz University, Jeddah, Saudi Arabia, where a medical education department (MED) was founded in 2007 in response to massive curricular changes and the introduction of a new undergraduate, integrated system-based curriculum in 1999 [2]. The MED now trains faculty in diverse fields and content through workshops and short courses on curriculum and course design, student assessment, teaching and learning strategies, and other educational areas.^9^ A retrospective study in 2015 analyzed the FDPs at the FOM at King Abdulaziz University from 2008 to 2014 and found that frequent workshops in the first three years (2008 to 2011) focused on students’ assessment. It is likely that the faculty’s participation in FDPs evolved according to their needs and major events in the institute or curriculum [2].

In 2007, the FOM implemented a new integrated system-based undergraduate curriculum. The introduction of different teaching strategies, mainly problem-based learning (PBL), necessitated an FDP to prepare faculty members as PBL facilitators, which was conducted and managed by the MED [16]. The program has been consistently offered since 2007 as a component of diverse faculty development initiatives, encompassing onboarding for new faculty members and ongoing training as required.This ongoing PBL-FDP has become mandatory for faculty members participating in PBL sessions at the FOM.

Specifically, the PBL-FDP aimed to introduce faculty members to the PBL process, facilitate learning, and evaluate PBL. Initially, it was scheduled for five days, with an introduction to the PBL process and discussions on the role and involvement of tutors and students in assessment and evaluation in PBL. In addition, two-day PBL simulation sessions were conducted. Later, considering the results of workshop evaluation surveys, the program was shortened to four and a half days, with only one simulation session [16]. These workshops sought to prepare faculty members with the knowledge and skills required for facilitating PBL and promote positive attitudes toward active learning through short presentations, scenario-based discussions, observations of role-modeled behavior, participant role-playing, and feedback [16].

In the context of King Abdulaziz University's Faculty of Medicine (KAU−FOM), undergraduate medical students in their third year are initially introduced to PBL within the curriculum's modular, integrated system−based approach. PBL tutorials are conducted in two−hour sessions, with groups comprising approximately 8–10 students convening three times per module.

Although the PBL-FDP has continued for nearly 10 years, there has been no comprehensive assessment of its outcomes. This assessment is important to confirm the program’s effectiveness and improve learning outcomes for both students and faculty members [16, 31]. Such an assessment would allow program organizers to measure the impact of the PBL-FDP, recognize areas for improvement, and make informed decisions for future revisions [24]. Moreover, it would provide valuable feedback to faculty members, allowing them to reflect on their teaching methods, identify their strengths and weaknesses, and make the necessary changes to enhance their PBL facilitation skills [5, 12].

One of the most widely used models for program assessment is the Kirkpatrick framework, which evaluates program outcomes across four levels (reaction, learning, behavior, and results) [23]. In 2016, this framework was systematically reviewed by [27] to investigate 111 studies on FDPs run to improve teaching effectiveness. They found that 50% of the studies evaluated reaction, 67% assessed participants’ learning, 81% assessed changes in behavior, and 26% assessed the program’s larger impact. Compared with their previous review [28], this review identified more diverse outcome levels in the literature and found the model suitable for evaluating the effectiveness of FDPs as well as changes in learners’ attitudes, knowledge, and skills.

While certain approaches in the literature have used pre- and post-surveying tools and direct observations of PBL, others have considered third-party perspectives such as students’ perceptions of faculty’s performance [11, 28]. Lim and Choy [17] employed the Kirkpatrick model to investigate the impact of a PBL tutor development program through student feedback,tutors’ confidence in teaching; and post-program surveys on changes in academic orientation, knowledge, and tutoring behaviors. Such an evaluation of outcomes allowed a structured investigation into the PBL program in terms of participants’ reactions, learning, and behavior and identified gaps in implementation.

Based on the foregoing, our research explored students’ evaluations of tutors and tutors’ insights into and experiences of PBL to inform the MED about necessary improvements to the existing PBL-FDP. This study evaluates the outcome of the PBL-FDP by integrating students’ feedback and tutors’ perceptions into the model, specifically its first (reaction) and second (learning) levels.

The first level of the model (reaction) measures participants’ immediate reactions to the training program. Previous research has demonstrated faculty members’ positive perceptions of PBL-FDPs, with the majority (88.8%) reporting being comfortable with the level of training received [16]. While gathering feedback from participants is a common method of assessing the first level of the model, using student feedback as a complementary source of information can provide valuable insights into the effectiveness of the program in terms of meeting the needs of students and improving the quality of education. Student feedback can also help identify areas in which PBL tutors can improve their performance by promoting different learning approaches. Students’ perspectives can thus provide a more objective assessment of the program’s effectiveness and help identify any biases or limitations in the feedback provided by the participants.

The second level of the Kirkpatrick model (learning) involves evaluating the learning outcomes of an FDP. Attitude is one of the three domains of learning outcomes, alongside knowledge and skills. Evaluating the attitude domain in the second level of the Kirkpatrick model involves assessing the extent to which the FDP has influenced tutors’ attitudes toward teaching. This can include tutors’ beliefs about the importance of promoting different learning approaches, their motivation to improve their teaching skills, and their confidence in their ability to facilitate tutorials.

To evaluate the attitude domain, this study uses the feedback collected from the students who participated in the PBL sessions led by the tutors in the FDP. Collecting feedback from students allows us to assess the extent to which the FDP has improved tutors’ ability to promote different learning approaches in PBL and change the program to better meet the needs of students. Furthermore, to understand tutors’ attitudes, this study also conducts focus group discussions with PBL tutors to identify their perceptions of the FDP and challenges they faced during the program.

## Methods

This mixed-methods study obtained ethical approval from the Unit of Biomedical Ethics on January 18, 2023. It was conducted in two phases. In phase one, the previously validated 13-item questionnaire presented by Dolmans and Ginns [10] was employed (see the Appendix). Over three months (January to March 2023), this was completed by 284 male and female third-year medical students at the FOM at King Abdulaziz University (male = 62.0%, *n* = 176, Table 1). Since the third year of the medical program at King Abdulaziz University is the first time that students experience PBL as a teaching strategy, the covering letter accompanying the questionnaire instructed them to evaluate all the tutors they had encountered during that academic year and think about the PBL courses as a whole, rather than identifying individual subjects, topics, or tutors when answering the questions. Under FOM policy, attending the PBL-FDP is mandatory for every faculty member involved in facilitating PBL sessions. This fact confirmed that all the tutors assessed by the students had participated in the PBL-FDP. The questionnaire was distributed through Google Forms, an online platform that allowed for convenient and efficient data collection. The survey was open for three months, providing ample time for the students to respond. At the end, an open-ended question on students’ recommendations for improving PBL was included.

**Table 1:** Socio-demographic characteristics of the study population (*N* = 284)

Characteristic	Number	%
Total	284	100.0
Gender		
Male	176	62.0
Female	108	38.0

The questionnaire asked for the students’ agreement with items in five categories of learning approaches: (1) constructive/active learning, (2) self-directed learning, (3) contextual learning, (4) collaborative learning, and (5) intra-personal behavior. Responses to each item were sought on a five-point Likert scale (1 = strongly disagree, 5 = strongly agree) [18]. The items of each category were as follows:

- *Constructive/active learning*
  - ◦ Summarizing what has been learnt in one’s own words
  - ◦ Searching for links between the issues discussed in the tutorial group
  - ◦ Understanding underlying mechanisms/theories
- *Self-directed learning*
  - ◦ Generating clear learning issues on one’s own
  - ◦ Searching for various resources on one’s own
- *Contextual learning*
  - ◦  Applying knowledge to the discussed problem
  - ◦  Applying knowledge to other situations/problems
- *Collaborative learning*
  - ◦ Giving constructive feedback on group work
  - ◦ Evaluating group cooperation regularly
- *Intra-personal behavior*
  - ◦ Providing a clear picture of their strengths and weaknesses as a tutor
  - ◦ Showing a clear motivation to fulfil the role of a tutor

Additionally, qualitative data were collected from a semi-structured focus group, consisting of three groups of 21 academic staff at the FOM who had attended PBL-FDPs over the past 10 years. The participants had wide-ranging academic experience, as they included both junior and senior members of the academic staff. The principal investigator, an expert in PBL, led all three groups. Each discussion lasted for 50–60 min and comprised open-ended questions to acquire deeper insights into the faculty members’ experiences and perspectives of PBL. The results of the phase one questionnaire were also discussed.

Owing to data saturation, only three focus group sessions were conducted. These were all recorded and subsequently transcribed, before being sent to the participants for checking. The resultant data were coded and grouped under five main themes: tutors’ insights into their strengths and weaknesses, challenges in conducting PBL, tutors’ ways of preparing for PBL, feedback, and suggestions for improving PBL workshops.

### Data analysis

The data obtained in phase one were analyzed and presented using IBM SPSS version 23 (IBM Corp., Armonk, N.Y., USA) and GraphPad Prism version 8 (GraphPad Software, Inc., San Diego, CA, USA). The required sample size was computed on the Raosoft website with an error margin of 5%, confidence level of 95%, population size of 415 faculty members, and response distribution of 50%. This suggested a minimum sample of 200 students. Of the 415 questionnaires distributed, 284 responses were collected (response rate = 68.4%). Hence, the number of responses exceeded the calculated minimum sample size. Numbers and percentages were used for the categorical variables, while the continuous variables were presented as means and standard deviations. Subsequently, a reliability analysis was conducted using Cronbach’s alpha values to study the properties, items, and average inter-item correlations of the measurement scales.

The correlations of the continuous variables were analyzed using Pearson’s correlation coefficients. The Chi-square test was used to establish the relationships among the categorical variables. Furthermore, to compare the means of two or more groups, an independent *t*-test was conducted under the assumption of a normal distribution. As an alternative test, Welch’s *t*-test was conducted. A *p*-value of < 0.05 was the criterion for rejecting the null hypothesis.

For the qualitative data analysis in phase two, the six steps of Braun and Clarke’s [7] framework for thematic analysis were followed:

#### Step 1: familiarizing with the data

The authors read and re-read transcripts of the recordings. Key ideas were marked and initial notes were made.

#### Step 2: generating initial codes

The data were organized systematically and meaningfully. Subsequently, each segment of data relevant to the research questions was open coded manually. The initial codes that emerged from reading the transcripts were discussed and preliminary codes were developed. All the researchers coded the transcripts separately and compared their codes later.

#### Step 3: searching for themes

After all the data were initially coded and collated, the analysis shifted to a larger scale by sorting the various codes into potential themes and collecting all relevant coded data extracts according to these themes. This resulted in a collection of candidate themes and sub-themes, and all data extracts coded in relation to them.

#### Step 4: reviewing themes

After collecting all the relevant data, their association with the themes was reviewed. The data sorted according to themes were coherent, meaningful, clear, and distinct.

#### Step 5: defining themes

The authors defined and further refined the themes. The three focus groups identified five themes: tutors’ insights into their strengths and weaknesses, challenges in conducting PBL, tutors’ ways of preparing for PBL, feedback, and suggestions for improving PBL workshops.

#### Step 6: writing up

The thematic analysis was reported.

## Results

**Fig. 1:**
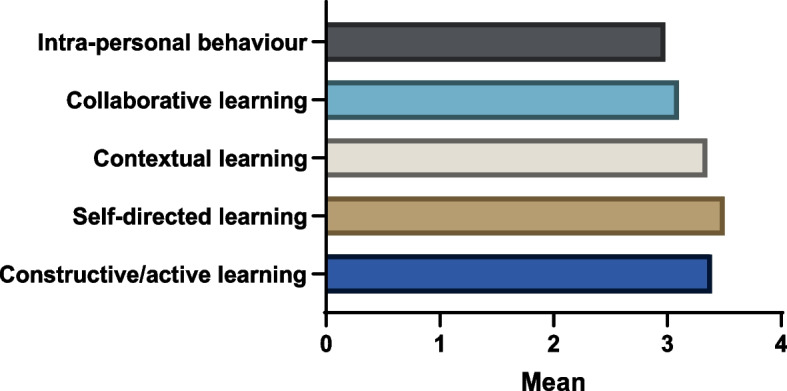
Mean scores of the learning approaches evaluated by the students (*N* = 284)

In the questionnaire, the 284 students evaluated tutors’ promotion of the five learning approaches. Table 2 presents the students’ levels of agreeability with those learning approaches. Nearly half agreed with the (a) “searching for links between the issues discussed in the tutorial group” (45.4%, *n* = 129), (b) “generating clear learning issues on one’s own” (40.5%, *n* = 115), (c) “searching for various resources on one’s own” (43.7%, *n* = 124), and (d) “applying knowledge to the discussed problem” (45.5%, *n* = 129) items. Furthermore, Table 3 shows that the self-directed learning category had the highest mean score of the learning approaches (mean ± SD = 3.50 ± 1.0, scale = 1–5), while the intra-personal behavior category had the lowest mean score (mean ± SD = 2.98 ± 1.0, scale = 1–5). At the item level, the “searching for various resources on one’s own” item had the highest mean score (mean ± SD = 3.58 ± 1.1, scale = 1–5), while the “providing a clear picture of their strengths and weaknesses as a tutor” item had lowest mean score (mean ± SD = 2.78 ± 1.2, scale = 1–5). Fig. 1 presents the mean scores of the learning approaches awarded by the students.

**Table 2:** Students’ levels of agreeability with the learning approaches of tutors (*N* = 284)

Variable	Strongly disagree	Disagree	Neutral	Agree	Strongly agree
***Constructive/active learning***					
Summarizing what has been learnt in one’s own words	24(8.5)	33(11.6)	77(27.1)	107(37.7)	43(15.1)
Searching for links between the issues discussed in the tutorial group	26(9.2)	28(9.9)	60(21.1)	129(45.4)	41(14.4)
Understanding underlying mechanisms/theories	25(8.8)	38(13.4)	84(29.6)	99(34.9)	38(13.4)
***Self-directed learning***					
Generating clear learning issues on one’s own	23(8.1)	36(12.7)	66(23.2)	115(40.5)	44(15.5)
Searching for various resources on one’s own	21(7.4)	27(9.5)	57(20.1)	124(43.7)	55(19.4)
***Contextual learning***					
Applying knowledge to the discussed problem	18(6.3)	26(9.2)	72(25.4)	129(45.4)	39(13.7)
Applying knowledge to other situations/problems	23(8.1)	49(17.3)	88(31.0)	100(35.2)	24(8.5)
***Collaborative learning***					
Giving constructive feedback on group work	34(12.0)	46(16.2)	88(31.0)	89(31.3)	27(9.5)
Evaluating group cooperation regularly	37(13.0)	48(16.9)	81(28.5)	85(29.9)	33(11.6)
***Intra-personal behavior***					
Providing a clear picture of their strengths and weaknesses as a tutor	54(19.0)	52(18.3)	104(36.6)	50(17.6)	24(8.5)
Showing a clear motivation to fulfil the role of a tutor	31(10.9)	39(13.7)	95(33.5)	85(29.9)	34(12.0)

**Table 3:** Mean scores of the learning approaches (*N* = 284)

Variable	Mean ± SD (1–5)
***Constructive/active learning***	***3.39* ± *1.0***
Summarizing what has been learnt in one’s own words	3.39 ± 1.1
Searching for links between the issues discussed in the tutorial group	3.46 ± 1.1
Understanding underlying mechanisms/theories	3.31 ± 1.1
***Self-directed learning***	***3.50* ± *1.0***
Generating clear learning issues on one’s own	3.43 ± 1.1
Searching for various resources on one’s own	3.58 ± 1.1
***Contextual learning***	***3.35* ± *0.9***
Applying knowledge to the discussed problem	3.51 ± 1.0
Applying knowledge to other situations/problems	3.19 ± 1.1
***Collaborative learning***	***3.10* ± *1.1***
Giving constructive feedback on group work	3.10 ± 1.2
Evaluating group cooperation regularly	3.10 ± 1.2
***Intra-personal behavior***	***2.98* ± *1.0***
Providing a clear picture of their strengths and weaknesses as a tutor	2.78 ± 1.2
Showing a clear motivation to fulfil the role of a tutor	3.18 ± 1.2

**Fig. 2:**
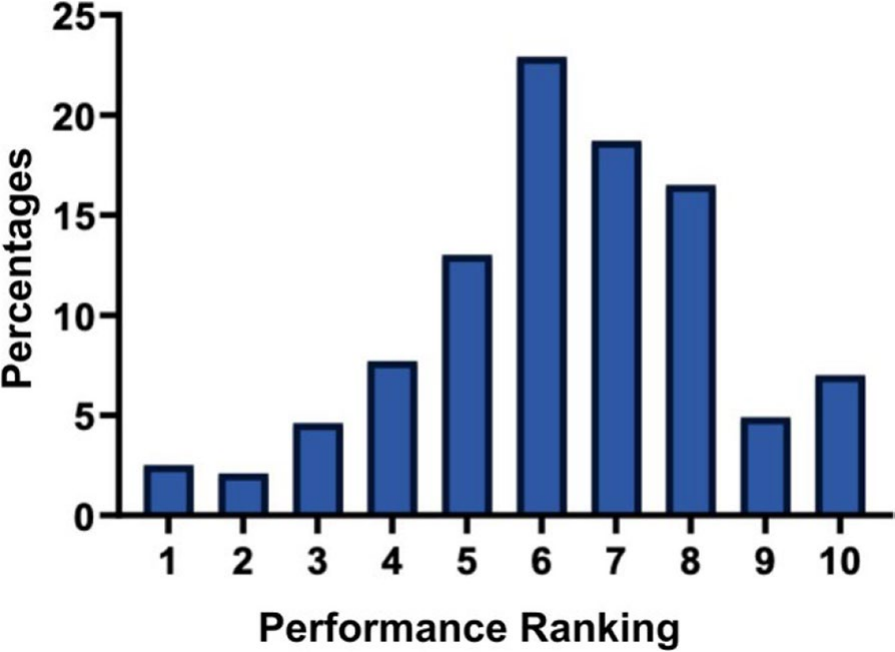
Mean performance rankings for tutors awarded by the students (*N* = 284)

Table 4 shows the mean performance ranking for tutors awarded by the students and tutors’ promotion of the learning approaches by percentage. The results reveal that the mean performance ranking for tutors was above halfway (mean ± SD = 6.32 ± 2.0, N = 284, min = 1, max = 10). The performance ranking was well distributed among rankings 5 (13.0%, *n* = 37), 6 (22.9%, *n* = 65), 7 (18.7%, *n* = 53) and 8 (16.5%, *n* = 47). Overall, most tutors performed sufficiently, namely they received rankings from 1 to 6 out of 10 (52.8%, *n* = 150). Fig. 2 presents the distribution of tutors’ mean performance rankings.

**Table 4:** Tutors’ mean performance ranking and promotion of the learning approaches (%; *N* = 284)

	**N**	**Min**	**Max**	**Mean**	**SD**
Performance ranking	284	1	10	6.32	2.0
		**Count**		**%**	
Total		284		100.0	
Performance ranking	1	7		2.5	
	2	6		2.1	
	3	13		4.6	
	4	22		7.7	
	5	37		13.0	
	6	65		22.9	
	7	53		18.7	
	8	47		16.5	
	9	14		4.9	
	10	20		7.0	
Performance ranking	Sufficient (1–6)	150		52.8	
	Excellent (7–10)	134		47.2

As presented in Table 5, the reliability statistics showed adequate Cronbach’s alpha values of 0.789 (*N* = 3, 95% CI = 0.74–0.83) for the constructive/active learning category, 0.608 (*N* = 2, 95% CI = 0.50–0.69) for the self-directed learning category, 0.710 (*N* = 2, 95% CI = 0.63–0.77) for the contextual learning category, 0.796 (*N* = 2, 95% CI = 0.74–0.84) for the collaborative learning category, and 0.738 (*N* = 2, 95% CI = 0.67–0.79) for the intra-personal behavior category.

**Table 5:** Reliability of the learning approach categories

Category	No. of items	Cronbach’s alpha	95% confidence interval
Constructive/active learning	3	0.789	0.74–0.83
Self-directed learning	2	0.608	0.50–0.69
Contextual learning	2	0.710	0.63–0.77
Collaborative learning	2	0.796	0.74–0.84
Intra-personal behavior	2	0.738	0.67–0.79

Table 6 presents the correlations among the learning approaches. The results reveal that the constructive/active learning category was significantly positively correlated (*N* = 284, *p* < 0.001) with all the other learning categories: self-directed learning (*r* = 0.503), contextual learning (*r* = 0.575), collaborative learning (*r* = 0.517), and intra-personal behavior (*r* = 0.604). This implies that when the constructive/active learning approach is adopted, the other learning approaches are established more dominantly. Similarly, the self-directed learning category was significantly positively correlated (*p* < 0.001) with the contextual learning (*r* = 0.509), collaborative learning (*r* = 0.338), and intra-personal behavior (*r* = 0.459) categories. The contextual learning category also had a significant positive correlation (*p* < 0.001) with collaborative learning (*r* = 0.555) and intra-personal behavior (*r* = 0.584). Finally, the collaborative learning and intra-personal behavior categories were significantly positively correlated (*p* < 0.001, *r* = 0.564).

**Table 6:** Correlations among the learning approaches

Category		Self-directed learning	Contextual learning	Collaborative learning	Intra-personal behavior
Constructive active learning	r	0.503^**^	0.575^**^	0.517^**^	0.604^**^
	*p*-value	< 0.001	< 0.001	< 0.001	< 0.001
	N	284	284	284	284
Self-directed learning	R		0.509^**^	0.338^**^	0.459^**^
	*p*-value		< 0.001	< 0.001	< 0.001
	N		284	284	284
Contextual learning	R			0.555^**^	0.584^**^
	*p*-value			< 0.001	< 0.001
	N			284	284
Collaborative learning	r				0.564^**^
	*p*-value				< 0.001
	N				284

^**^Correlation is significant at the 0.01 level (two-tailed)

Table 7 shows the associations for the male and female students’ assessments of tutors’ learning approaches. The results reveal significant associations among the scores for the constructive/active learning (female: 3.56 ± 0.9 vs. male: 3.28 ± 1.0, *p* = 0.019), self-directed learning (3.79 ± 0.9 vs. 3.33 ± 1.0, *p* < 0.001), contextual learning (3.55 ± 0.9 vs. 3.23 ± 0.9, *p* = 0.005), and collaborative learning (3.28 ± 1.1 vs. 2.99 ± 1.1, *p* = 0.031) approaches. Specifically, significantly higher scores were recorded among the female students, suggesting that tutors’ learning approaches tended to be viewed more favorably by this group. Moreover, the female students awarded tutors the highest score for the self-directed learning approach (3.79 ± 0.9), while the male students awarded the lowest score for the intra-personal behavior approach (2.97 ± 1.1).

**Table 7:** Associations between the male and female students’ assessment of tutors’ learning approaches

Category	Male	Female	*p*-value
Total	176	108	-
Constructive/active learning	3.28 ± 1.0	3.56 ± 0.9	0.019^a^
Self-directed learning	3.33 ± 1.0	3.79 ± 0.9	< 0.001^a^
Contextual learning	3.23 ± 0.9	3.55 ± 0.9	0.005^a^
Collaborative learning	2.99 ± 1.1	3.28 ± 1.1	0.031^a^
Intra-personal behavior	2.97 ± 1.1	3.00 ± 0.9	0.824

^a^Significant using the independent *t*-test at < 0.05

Table 8 presents the associations between the sufficient and excellent scores for tutors’ learning approaches awarded by the students. The independent *t*-test and Welch’s *t*-test results reveal significant associations (*p* < 0.001) among all the learning approaches. Specifically, significantly higher excellent scores than sufficient scores were reported for the constructive/active learning (excellent: 3.87 ± 0.7 vs. sufficient: 2.95 ± 0.9), self-directed learning (3.93 ± 0.7 vs. 3.12 ± 1.0), contextual learning (3.80 ± 0.7 vs. 2.95 ± 0.9), collaborative learning (3.59 ± 0.9 vs. 2.66 ± 1.0), and intra-personal behavior (3.56 ± 0.8 vs. 2.47 ± 1.0) approaches.

**Table 8:** Associations between the sufficient and excellent scores for tutors’ learning approaches awarded by the students

Category	Sufficient	Excellent	*p*-value
Total	150	134	-
Constructive/active learning	2.95 ± 0.9	3.87 ± 0.7	< 0.001^b^
Self-directed learning	3.12 ± 1.0	3.93 ± 0.7	< 0.001^b^
Contextual learning	2.95 ± 0.9	3.80 ± 0.7	< 0.001^b^
Collaborative learning	2.66 ± 1.0	3.59 ± 0.9	< 0.001^a^
Intra-personal behavior	2.47 ± 1.0	3.56 ± 0.8	< 0.001^b^

^a^Significant using the independent *t*-test at < 0.05

^b^Significant using Welch’s *t*-test at < 0.05

Table 9 shows the correlations between the students’ performance rankings for their tutors and the learning approaches. The results reveal a significantly positive correlation between tutors’ performance rankings and all the learning approaches: constructive/active learning (*p* < 0.001, r = 0.640), self-directed learning (*p* < 0.001, *r* = 0.537), contextual learning (*p* < 0.001, *r* = 0.568), collaborative learning (*p* < 0.001, *r* = 0.638), and intra-personal behavior (*p* < 0.001, *r* = 0.638). This suggests that students award tutors higher performance rankings when there is greater engagement with learning approaches.

**Table 9:** Correlations between the performance ranking for tutors awarded by the students and learning approaches

Category		Performance ranking (1–10)
Constructive/active learning	r	0.640^**^
	*p*-value	< 0.001
	N	284
Self-directed learning	r	0.537^**^
	*p*-value	< 0.001
	N	284
Contextual learning	r	0.568^**^
	*p*-value	< 0.001
	N	284
Collaborative learning	r	0.472^**^
	*p*-value	< 0.001
	N	284
Intra-personal behavior	r	0.638^**^
	*p*-value	< 0.001
	N	284

^**^Correlation is significant at the 0.01 level (two-tailed)

Finally, Table 10 presents the associations between the students’ gender and tutors’ mean performance ranking. The results show a significant association (*p* = 0.001) between the students’ gender (both male and female) and their provision of sufficient and excellent performance rankings for their tutors. Specifically, significantly higher sufficient and excellent rankings were observed among the male students [sufficient: 71.3% (*N* = 107) vs. 28.7% (*N* = 43)] than among the female students [excellent: 51.5% (*N* = 69) vs. 48.5% (*N* = 65)]. However, the female students (6.87 ± 1.9) awarded a significantly higher mean performance ranking (1–10) than the male students (5.98 ± 2.1) (*p* < 0.001), according to the independent *t*-test at < 0.05.

**Table 10:** Associations between the students’ gender and tutors’ mean performance ranking (N = 284)

		**Total**	**Gender**		***p*-value**
			**Male**	**Female**	
Total		284	176 (62.0%)	108 (38.0%)	-
Performance ranking	Sufficient	150	107 (71.3%)	43 (28.7%)	0.001^a^
	Excellent	134	69 (51.5%)	65 (48.5%)	
	**Mean ± SD (1–10)**		5.98 ± 2.1	6.87 ± 1.9	< 0.001^b^

^a^Significant using the Chi-square test at < 0.05

^b^Significant using the independent *t*-test at < 0.05

At the end of the questionnaire, the students were asked an open-ended question on their recommendations for improving PBL. The following five themes emerged from their responses.

### Tutor’s role

Some students opined that during the sessions, the tutor should be more of a facilitator than an observer. Moreover, the tutor should put effort into preparing for the sessions. Some preferred having a tutor who specialized in the same area as the discussed case to gain more knowledge about the topic. However, others believed that tutors should provide them with information and resources.

### PBL session planning

Some students suggested conducting online PBL sessions. Many of them complained about the timing of PBL within the module and suggested separating PBL sessions from exams. Other concerns were the inclusion of materials that required explanation through lectures and topic redundancy, as students felt that the same information was repeated in lectures and PBL sessions. Thus, they suggested that the topics and materials taught in lectures be integrated and supported by PBL. Additionally, students emphasized that cases should be clear, relevant, and meaningful, for a comprehensive understanding of the purpose of the case. Moreover, they thought that certain objectives were difficult, vast, unclear, and scattered across the various PBL groups. They suggested re-evaluating the objectives of PBL cases.

### PBL implementation

Some students preferred to use audio-visual material for enhancing learning. Others reported that PBL sessions were misused as homework or for completing presentations and assignments. They also felt the need for clear points for evaluation.

### Safe learning environment

Students shared that in certain sessions, they did not feel safe to express their thoughts and ideas, without the fear of harsh criticism.

### Improving group dynamics

The students opined that participation should be encouraged to make the sessions more motivational and interactive.

The data from the faculty focus groups were also analyzed according to the five major themes: tutors’ insights into their strengths and weaknesses, challenges in conducting PBL, tutors’ ways of preparing for PBL, feedback, and suggestions for improving PBL workshops.

### Theme 1: tutors’ insights into their strengths and weaknesses

Most of the senior faculty members said that at the beginning of their tutoring journey, they would reflect upon their practice, strengths, and weaknesses after each session, until they mastered them and it became routine. Overall, the participants cited both direct and indirect ways of gaining greater insight into one’s practices. The group agreed that receiving direct feedback from students helped identify their strengths and weaknesses. An additional indirect method was observing group dynamics, such as students’ motivation, interaction, and engagement. They added that reflection and improvement are dynamic processes. They explained that, for example, if students performed well, it made a tutor believe that they too were doing well. The group also mentioned that by reading and expanding knowledge and mindfulness, they could assess their strengths and weaknesses better.

### Theme 2: challenges in conducting PBL

#### Tutor-related challenges

Time management was a major challenge, followed by the acceptance of diversity among students and management of dominant students. The age gap between the tutor and students was cited as another challenge in PBL. The other challenges mentioned were meeting objectives and obtaining answers from students. Additionally, some of the tutors perceived case content as challenging, especially when they found the case stimulating. If a tutor does not prefer the case’s specialty or context, then facilitating and making students view the case as a real-world scenario become challenging. Lastly, an emerging challenge was the non-recognition of the value of PBL by some of the tutors, who were only looking to cover their teaching hours.

#### Student-related challenges

Most of the faculty members faced challenges in convincing students of the value of PBL. Another challenge was involving students, as they seemed unmotivated, exhausted, and uncommitted to achieving the objectives. The tutors added that students tended to distribute the objectives among themselves, causing difficulties in summarizing and limiting the diversity of research resources.

### Theme 3: tutors’ ways of preparing for PBL

#### Pre-session preparation

Before the session, most of the faculty members reviewed the curriculum, lectures, and objectives. However, some only read the objectives; their intention was to remain authentic by considering themselves as students and ignite interest in investigating the case. They believed that this would make students feel more responsible for their learning processes.

#### Case-related preparation

The group agreed that creating cases on the basis of real-world scenarios helped prepare the sessions. Most of the faculty members thought that the best ways to prepare were reading the case in advance, reviewing the objectives, and devising ways to direct, stimulate, and engage students. Another approach was watching videos of PBL sessions, reading the latest reviews of the case, and searching for advanced resources. In case discussion sessions, some of the tutors addressed clarifications by case writers to understand how to link the case objectives.

#### Site-related preparation

One group mentioned that they usually did not check the assigned session locations beforehand, while another group would check the location for suitability.

### Theme 4: feedback

The tutors approached feedback differently. Some of the faculty members provided group feedback at the end of each session by collectively asking students about what went well and what needed improvement in terms of the sessions, cases, and tutor. Others provided feedback only on the PBL content and process. Only one of the 21 tutors participating in the focus groups provided individual feedback to each student; the others did not do this because of time constraints. Some of the tutors allowed students to provide peer feedback. However, they agreed that students were not trained to do this. Overall, there was consensus on feedback being an integral part of the PBL process.

### Theme 5: suggestions for improving PBL workshops

When questioned about the prospect of being observed by a trained PBL faculty member from the MED during sessions and receiving reports on their strengths and possible areas of improvement, the senior tutors were supportive but only as an optional opportunity outside the purview of administration. By contrast, the junior tutors felt that observations might be stressful and instead suggested internal peer review within departments.

## Discussion

The results from phase one of our study showed that the mean performance ranking for tutors awarded by the students was above halfway (mean ± SD = 6.32 ± 2.0, *N* = 284, min = 1, max = 10) and that a significantly positive correlation existed among tutors’ performance rankings for all five learning approaches (p < 0.05). These findings contradict those of a similar study conducted et al.-Imam Mohammad Ibn Saud Islamic University, which used a questionnaire to investigate students’ perceptions of the conduct, processes, and benefits of PBL sessions. That questionnaire included three items on students’ perceptions of tutors’ facilitation and fairness in PBL [4]. The study found that only 28.3% and 26.3% of the students, respectively, agreed that tutors were well prepared for the sessions and evaluated fairly. Based on these results, a need for more intensive faculty training was concluded [4]. However, our study found that the constructive/active learning approach exhibited a significant positive correlation with the other learning approaches, suggesting that the more frequently it is demonstrated, the more pertinently are the other approaches established among tutors.

Another study found that tutors must develop the abilities and attitudes necessary for “supporting the learning process and metacognitive knowledge” [30]. Our results also suggest the need for PBL-FDPs, as both the intra-personal behavior category and the “providing a clear picture of their strengths and weaknesses as a tutor” item received the lowest scores. Incorporating peer coaching and tutor shadowing, followed by reflection and feedback on PBL-FDPs, is recommended to overcome these challenges. According to Johnson, interactions between tutors help disseminate and improve best practices as well as reflect on learning experiences [15]. Tsai et al. [29] evaluated the effectiveness of tutor shadowing on faculty development in PBL, using a pre- and post-shadowing activity rating scale to measure novice tutors’ self-rated confidence in several domains. They found that tutor shadowing was effective in improving their self-rated confidence.

The constructive/active learning category represents a tutor’s level of understanding and suitable implementation of the steps and strategies of PBL. This conceptual knowledge is always the first level of any faculty development or tutor training program for PBL [22]. Our findings demonstrate the importance of valuing and advancing this category in PBL workshops. This is similar to that demonstrated by Irby’s classification in 1996, which he used to explain the progress of most PBL development programs for medical faculty [14]. First, faculty members were presented with the concept and value of PBL. They were then introduced to the general knowledge and skills relevant to their role as tutors as well as to specific knowledge tailored to suit the content they taught. The study also introduced five models of FDPs in PBL: a general skills model, general skills plus model, developmental model, comprehensive model, and course-based model. Irby argued that such a progressive implementation of PBL-FDPs would help faculty members develop high-level problem-solving and analytical skills, which would further nurture leadership and scholarship skills. With reference to this classification, the FOM at King Abdulaziz University follows the general skills plus model, as it discusses the general concept of PBL, role of tutors, evaluation, and feedback. Efforts should be made to advance workshops and incorporate an expanded PBL-FDP model.

This study also revealed that the greater the tutor’s engagement, the stronger the students’ tendency to award them higher performance rankings. This is similar to [33] suggestion that tutors should engage with students, provide feedback, and be open to reflecting on their own practice to improve their teaching. These findings emphasize the importance of tutors being skilled in various approaches to promote PBL among students. This also necessitates that the MED deliberately plans FDPs to equip FOM faculty members with wide-ranging skills for helping students achieve learning outcomes. The current FDPs at the FOM must be examined to determine whether this intention exists. Based on our findings from phase two of the study and participants’ inputs, there are three recommendations for improving the existing PBL-FDP.

### Recommendations for implementing PBL

To improve the implementation of the PBL-FDP, it is recommended that training in providing constructive, scientific feedback during PBL sessions be conducted. Most of the tutors in our study preferred direct and indirect feedback to gain deeper insights into their practice and thus did not comprehend the importance of providing constant feedback to students in PBL tutorials. The availability of a feedback rubric for use in PBL sessions may be useful. Pangastuti et al. [21] introduced a feedback model for PBL tutorials and found that it had a positive impact on cognitive and behavioral changes among both students and tutors. Most faculty members recommended initiating a student orientation program to raise awareness of PBL values, processes, roles, and tutors’ responsibilities. Additionally, during our focus group discussion, the participants expressed openness to receiving direct feedback from an expert observer to improve performance or engage in a simulated PBL session with an expert tutor, which would allow for open discussions. This skill enhancement initiative can be incorporated into faculty improvement initiatives and program outcome evaluations.

Garcia et al. [12] used peer and self-observation of video-tabbed PBL sessions, supported by an assessment rubric and hour-long feedback session, as approaches to self-reflection, tutors’ skill enhancement, and program evaluation. The results showed that these were effective in raising the participants’ awareness of their strengths and weaknesses, motivating them to improve practice, and making them more eager to learn. This sort of program evaluation strategy made it possible to identify tutors’ needs and the roots of their challenges, which in turn served as the foundation for further improvement. The participants also recommended access to online learning resources such as videos, recordings, and manuals to enhance tutors’ self-learning and ensure continuous improvement. This is similar to Johnson’s [15] recommendations of training PBL tutors, using active learning strategies such as role-playing and case studies and engaging tutors in the learning process so they can practice and apply their skills. The author also recommended providing continuous support and resources to tutors through mentorship programs, online resources, and collaboration and networking opportunities,furthermore, emphasis was placed on the continuous professional development of PBL tutors to ensure that they remained up-to-date with the latest research and best practices in PBL [21].

### Logistical recommendations

Logistical matters, including timetables and physical aspects (e.g., lighting, ventilation, and classroom seating arrangements), are important factors for the success of PBL [18]. The existing literature illustrates that since PBL is a “curricular structure more than a pedagogy,” its successful implementation requires several factors such as infrastructure, human resources, and logistics [6]. Vogt et al. [33] provided valuable insights into the logistical aspects of PBL implementation and factors that influence its success. The authors described the challenges to implementing PBL, including the time- and resource-intensive nature of the approach. They argued that institutions must support PBL implementation through adequate resources such as dedicated personnel, funding for learning material, and convenient administrative policies. They also discussed the importance of technology in PBL implementation, such as the use of online resources and virtual learning environments.^28^ The authors recommended collaboration among faculty members, continuous evaluation and feedback, and institutional support. By following these recommendations, institutions can ensure that the PBL approach is implemented effectively and that students receive a high-quality learning experience that is engaging, interactive, and effective [33]. Moreover, the development of students’ cognitive skills, especially problem-solving, decision-making, and critical thinking, requires them to be attentive and energetic. Therefore, PBL sessions should be scheduled either as early as possible or immediately after examinations. A study conducted at the University of Sharjah, United Arab Emirates investigated the effectiveness of PBL in the medical curriculum by surveying medical students’ perceptions of PBL and its impact on learning outcomes [3]. It found that some students were concerned about the heavy workload and time-consuming nature of PBL. The authors suggested careful planning and support to ensure that students manage their workload effectively. They opined that the benefits of PBL such as improved critical thinking and problem-solving skills outweigh the challenges associated with a heavy workload.

### Recommendation for quality and development units

The students and tutors agreed on the need for student feedback after each PBL session. Dolmans et al. [9] discussed the role of tutors and importance of feedback in PBL. They argued that feedback is essential for students to develop problem-solving skills and refine their understanding of a subject matter. The authors also suggested giving students the opportunity to provide feedback on PBL sessions’ quality and tutors’ performance. Such feedback can make the PBL process more effective and enhance students’ learning experiences [9]. In our context, the quality department can create a student feedback mechanism that focuses on the learning process and tutors’ skills. As a teaching strategy, specific questions on PBL should be included either in a general course or curriculum evaluation. Systematic and official tutor evaluations need to be collected and analyzed, and specific reports should be sent to each tutor for self-improvement. A collective copy of the challenges faced by PBL tutors should be sent to the MED, so that specific PBL-FDPs may be formed for them. It is also recommended that quality and development support bodies fairly standardize the assessment of students in PBL. Such matters have been comprehensively reinforced and studied in diverse publications,for instance, Des Marchais and Vu [8] proposed a standardized system for assessing students in PBL. They explained its design and development by incorporating various methods such as written exams, portfolios, and oral presentations and integrating them into the curriculum. Their results showed that the assessment system was effective at measuring students’ learning outcomes and promoting critical thinking. The authors concluded that this assessment system could serve as a model for institutions seeking to implement PBL curricula with effective assessment strategies.

### Limitations

First, this study focused only on one student group, that is, third-year students at the FOM at King Abdulaziz University, whose experiences cannot be generalized to other institutions in the country. Therefore, the study may not be representative of all medical colleges in Saudi Arabia. Future studies could extend this model to multiple centers to provide a clear image of PBL-FDPs in Saudi Arabia. Second, the study focused on the perceptions of those students attending PBL sessions. Future studies could involve students who had previously engaged with PBL. Third, the study did not investigate the characteristics of individual tutors that may have influenced the evaluation by the students. This study design does not allow for isolating the sole effect of the FDP on tutors attitude and students' feedback. Other factors, such as the duration of PBL experience and participation in other professional development programs, may have influenced the results Future research should employ a more robust design to isolate the specific effects of the FDP. Future research could consider exploring the impact of individual tutor characteristics such as their level of experience, the year in which they attended the PBL-FDP, their teaching style, and other relevant factors on the evaluation. By investigating the various attributes of individual tutors, future studies could gain a better understanding of the factors that help facilitate PBL-FDPs. This understanding could help develop tailored FDPs that address the needs of individual tutors and enhance their facilitation skills. Fourth, we applied the first level and part of the second level (attitude) of the Kirkpatrick model for the evaluation. Further studies are required to gather data at the third and fourth evaluation levels. Future research could consider investigating the impact of FDPs on faculty members’ teaching practices and student learning outcomes in the other domains of learning outcomes (knowledge and skills). Furthermore, studies could explore the effectiveness of different training models, duration and intensity of training, and use of innovative training methods on student satisfaction with tutor facilitation. Additionally, focus groups where students discuss and confirm the results can be conducted to support the evidence. Finally, studies could investigate the long-term impact of FDPs on faculty members’ professional development and the sustainability of the culture of continuous improvement and professional growth.

## Conclusions

It is important to consider student feedback when planning FDPs, as it provides valuable insights into students’ experience and improves the effectiveness of PBL. Student feedback is a fundamental source of information; it provides tutors with a deep understanding of their facilitation practices and allows areas for improvement to be identified. By actively involving students, the MED can align and tailor programs to address specific challenges and the goals of the primary beneficiaries—the students. In conclusion, conducting PBL-FDPs with focus on the challenges faced by faculty members can both improve the overall teaching and learning process and directly enhance students’ learning outcomes. These programs provide faculty members with a platform for developing facilitation skills, discovering innovative instructional strategies, and addressing the diverse challenges encountered in classrooms. Faculty members have the opportunity to collaborate, share experiences, and learn from each other. By creating a supportive community of educators, FDPs can foster a culture of continuous improvement and professional growth.

### Supplementary Information

Supplementary Material 1.

## Data Availability

The datasets used and/or analyzed during the current study are available from the corresponding author on reasonable request.

## Abbreviations

FOM: Faculty of Medicine
FDPs: Faculty development programs
PBL: Problem-based learning
MED: Medical education department
