# Supplementary material for: Employing students’ evaluations and tutors’ perceptions to evaluate a faculty development program on problem-based learning at the Faculty of Medicine, King Abdulaziz University
Source: BMC Med Educ. 2024 Jul 1;24:708. doi: 10.1186/s12909-024-05662-1 (PMC11218292; doi:10.1186/s12909-024-05662-1)
Supplement: Supplementary file 1 — Supplementary Material 1. [file 12909_2024_5662_MOESM1_ESM.zip › Stage200/author/12909_2024_5662_MOESM1_ESM.docx]

**Appendix**

**Tutor evaluation questionnaire**

**(Covering letter)**

In the medical education department, we are evaluating the problem-based learning (PBL) process in our Faculty of Medicine to help it meet its educational goals. This questionnaire is designed to garner students’ perspectives of their PBL tutors. For the following statements, indicate how much you agree with each on a scale from 1 to 5 (1 = strongly disagree, 5 = strongly agree). While answering this questionnaire, please think about the PBL courses as a whole rather than identifying individual subjects, topics, or tutors. All data will remain confidential.

| Constructive/active learning | |
| --- | --- |
| 1 | The tutor encouraged us to summarize what we had learnt in our own words |
| 2 | The tutor encouraged us to search for links between the issues discussed in the tutorial group |
| 3 | The tutor encouraged us to understand underlying mechanisms/theories |
| Self-directed learning | |
| 4 | The tutor encouraged us to generate clear learning issues by ourselves |
| 5 | The tutor encouraged us to search for various resources by ourselves |
| Contextual learning | |
| 6 | The tutor encouraged us to apply knowledge to the discussed problem |
| 7 | The tutor encouraged us to apply knowledge to other situations/problems |
| Collaborative learning | |
| 8 | The tutor encouraged us to give constructive feedback on group work |
| 9 | The tutor encouraged us to evaluate group cooperation regularly |
| Intra-personal behaviour | |
| 10 | The tutor had a clear picture of their strengths and weaknesses as a tutor |
| 11 | The tutor was clearly motivated to fulfil their role as a tutor |
|  |  |
| Performance ranking | |
| 12 | Give a ranking (1–10) for the performance of the tutor (6 being sufficient, 10 being excellent) |
| Open-ended question | |
| 13 | Give the tutor tips for improvement. Do this especially if you gave your tutor a score below 6. |
