# Supplementary figures and images for: Employing students’ evaluations and tutors’ perceptions to evaluate a faculty development program on problem-based learning at the Faculty of Medicine, King Abdulaziz University
Source: BMC Med Educ. 2024 Jul 1;24:708. doi: 10.1186/s12909-024-05662-1 (PMC11218292; doi:10.1186/s12909-024-05662-1)

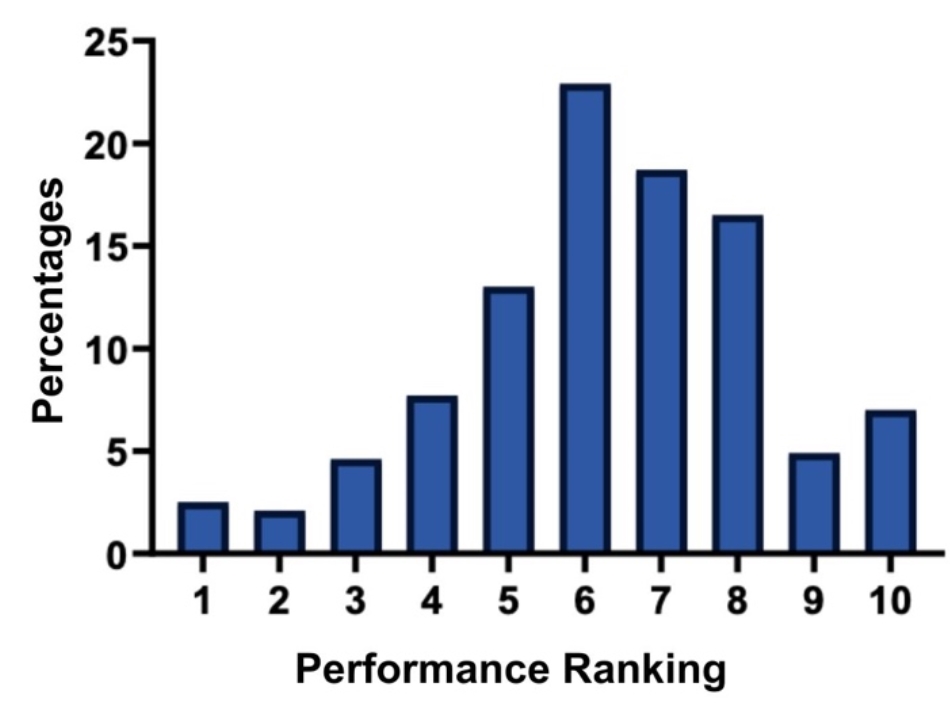

Supplement: Supplementary file 1 — Supplementary Material 1. [file 12909_2024_5662_MOESM1_ESM.zip › Stage200/author/12909_2024_5662_Fig2_Print.jpeg]

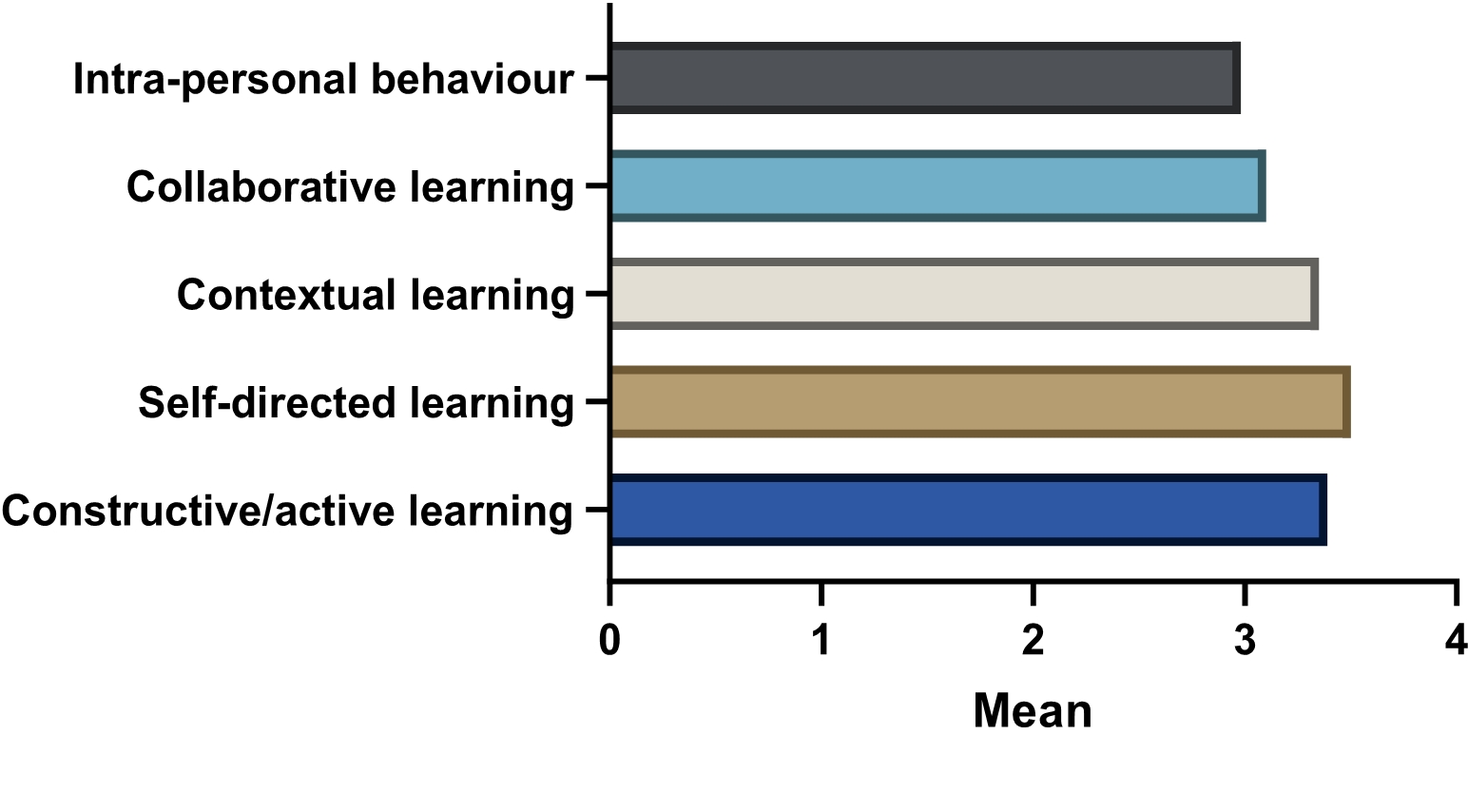

Supplement: Supplementary file 1 — Supplementary Material 1. [file 12909_2024_5662_MOESM1_ESM.zip › Stage200/author/12909_2024_5662_Fig1_Print.jpeg]

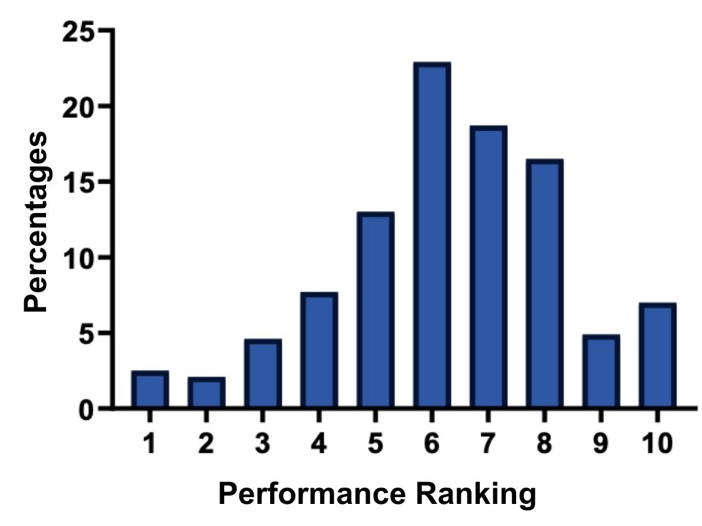

Supplement: Supplementary file 1 — Supplementary Material 1. [file 12909_2024_5662_MOESM1_ESM.zip › Stage200/author/Figures/Figure_2.docx]
